# Non-coding RNA and its network in the pathogenesis of Myasthenia Gravis

**DOI:** 10.3389/fmolb.2024.1388476

**Published:** 2024-09-10

**Authors:** Fuqiang Wang, Xiaoli Mei, Yunhao Yang, Hanlu Zhang, Zhiyang Li, Lei Zhu, Senyi Deng, Yun Wang

**Affiliations:** ^1^ Department of Thoracic Surgery, West China Hospital of Sichuan University, Chengdu, China; ^2^ Department of Thoracic Surgery, Institute of Thoracic Oncology, West China Hospital, Sichuan University, Chengdu, China; ^3^ Department of Thoracic Surgery, West China Hospital, West China School of Nursing, Sichuan University, Chengdu, China

**Keywords:** autoimmune disease, Myasthenia Gravis, non-coding RNA, ceRNA, regulatory network

## Abstract

Myasthenia Gravis (MG) is a chronic autoimmune disease that primarily affects the neuromuscular junction, leading to muscle weakness in patients with this condition. Previous studies have identified several dysfunctions in thymus and peripheral blood mononuclear cells (PBMCs), such as the formation of ectopic germinal centers in the thymus and an imbalance of peripheral T helper cells and regulatory T cells, that contribute to the initiation and development of MG. Recent evidences suggest that noncoding RNA, including miRNA, lncRNA and circRNA may play a significant role in MG progression. Additionally, the network between these noncoding RNAs, such as the competing endogenous RNA regulatory network, has been found to be involved in MG progression. In this review, we summarized the roles of miRNA, lncRNA, and circRNA, highlighted their potential application as biomarkers in diagnosing MG, and discussed their potential regulatory networks in the abnormal thymus and PBMCs during MG development.

## 1 Introduction

Myasthenia Gravis (MG) is a chronic autoimmune disease that mainly affects the neuromuscular junction (NMJ), leading to weakness of patients’ muscles (Gilhus, 2016; El-Salem et al., 2014). MG can be classified into several subtypes according to the age of disease onset, types of autoantibodies and clinical manifestation (El-Salem et al., 2014). Based on the age of disease onset, the MG is classified into early onset MG (EOMG, ≦ 50-year-old) and late onset MG (LOMG, >50-year-old) (Szczudlik et al., 2014; Huang et al., 2023). The EOMG is more frequent in young women with thymic hyperplasia and LOMG usually occurs in middle-aged and elderly men with thymoma (Berrih-Aknin and Le Panse, 2014). Depending on the type of serum autoantibodies, MG can generally be classified into acetylcholine receptor antibody positive MG (AChR^+^ MG), muscle-specific tyrosine kinase antibody positive MG (MuSK^+^ MG), or lipoprotein receptor-related protein 4 antibody positive MG (LRP4^+^ MG) (Huang et al., 2023). Among them, AChR^+^ MG is the most frequent subgroup of MG. In some cases, other autoantibodies such as Titin antibody, or ryanodine receptor antibody (RyR-Ab) can also be detected in patients’ serum (Skeie et al., 2006). Based on the clinical manifestation of MG, in other words, depending on the type of muscle involved, MG can be classified into ocular MG (OMG) and generalized MG (GMG) (Monsul et al., 2004). The ocular muscle is typically the initial affected muscle, resulting in ptosis and diplopia, which are usually the first symptoms of MG. Gradually, as the muscles of the limbs and trunk are affected, patients demonstrate limb weakness and may also experience dysarthria and dysphagia (bulbar symptoms), as well as respiratory difficulties. This condition is known as generalized MG. Due to the different characteristic of MG subtypes, the mechanism behind the different types of MG is variable (El-Salem et al., 2014).

Although muscle is the primary target organ of MG, the effector organ is generally considered to be the thymus. Thymic lesions, including thymic hyperplasia and thymoma, play crucial roles in the pathological process of AChR^+^ MG (Cron et al., 2020). The thymus serves as the organ for T cell maturation. Physiologically, naive T cells can only survive and mature after positive and negative selection in cortical and medullary thymus, respectively (Sprent and Kishimoto, 2002; Ashby and Hogquist, 2024). Positive selection secures T cells with ability to bind MHC complexes and negative selection secures T cells without autoimmune potential (Ashby and Hogquist, 2024). However, for MG patients with thymoma, the negative selection of thymus is largely compromised (Berrih-Aknin and Le Panse, 2014). Tumor cells of thymoma, derived from the cortical thymic epithelial cells (cTECs), lack medullary thymic epithelial cells (mTECs). Moreover, the decreased expression of autoimmune regulator (AIRE) and forkhead box protein P3 (FOPX3), which play a crucial role in preventing autoimmune responses, can also be observed in thymoma (Berrih-Aknin and Le Panse, 2014). As a result, T cells with autoimmune potential may not be deleted effectively in the thymoma of MG patients due to the lack of negative selection. The pathologic mechanism of thymic hyperplasia is distinct from thymoma in MG. The ectopic germinal centers (GCs) formed by numerous B cells could secrete AChR antibodies (Berrih-Aknin and Le Panse, 2014). Moreover, the ectopic high endothelial venules (HEVs) and chemokines such as CXCL13 and CCL21 could recruit the pro-inflammatory cells in thymus, activate inflammation, and compromise the normal immune regulatory process (Berrih-Aknin and Le Panse, 2014).

Although the mechanisms underlying thymoma and hyperplastic thymus-induced MG differ, noncoding RNAs including micro RNA (miRNA), long noncoding RNA (lncRNA) and circular RNA (circRNA), play crucial roles in both processes (Cron et al., 2020). Generally, these noncoding RNAs cannot encode protein, but it can regulate the expression of mRNA through posttranscriptional modification (Cai et al., 2009). MiRNA is short (approximately 20–25 nucleotides) single-stranded noncoding RNA, lncRNA is noncoding RNAs longer than 200 nucleotides, and circRNA is circular non-coding RNA. As important part of epigenetics, increasing evidence shows that noncoding RNA is important in the pathological process of MG (Berrih-Aknin and Le Panse, 2014). Moreover, benefiting from the easy availability and circulating stability of ncRNA, researchers screen out several ncRNAs for diagnosing MG and predicting the effectiveness of treatment for MG (Punga and Punga, 2018; Yoganathan et al., 2022). Newly identified non-coding RNAs, such as rRNA-derived small RNAs and tRNA-derived small RNAs, have also been found to play regulatory roles in various diseases (Zhang et al., 2023; You et al., 2019). However, these non-coding RNAs have not been extensively studied in MG. In this paper, we reviewed the roles of miRNA, lncRNA, and circRNA and their networks that participating in the pathomechanism of MG and summarized the ncRNA which may act as potential biomarkers for MG.

## 2 The role of miRNA in diagnosing and pathogenesis of MG

MiRNAs are short (approximately 20–25 nucleotides) single-stranded RNA molecules (Blow et al., 2006). Mature miRNAs can base pair with target mRNAs, post-transcriptionally inhibiting mRNA expression through the RNA-induced silencing complex (Gregory et al., 2005). These miRNAs play significant roles in both physiological and pathological processes within the human body, including immunity, inflammation, and oncogenesis (Singh et al., 2013; Zhang et al., 2007). Notably, miRNAs exhibit stable expression in peripheral blood, facilitated by vesicles and protein complexes (Kai et al., 2018; Cheng et al., 2014). Consequently, miRNAs hold promise as potential diagnostic biomarkers for assessing and predicting treatment responses in MG (Huang et al., 2023; Punga and Punga, 2018; Sabre et al., 2020). In this review, we summarize existing research on miRNA profiles and their diagnostic potential in MG.

### 2.1 miRNA as a potential biomarker of MG

The MG related autoantibody is currently the specific tool for diagnosing MG. However, the titer of autoantibody cannot be used to predict therapy response of MG. In recent years, due to their ease of detection and stability in serum, miRNAs have increasingly been recognized as novel biomarkers of diseases diagnosis (Kai et al., 2018). In MG patients, dysregulated miRNAs can be released into peripheral blood from thymic tissue or peripheral blood mononuclear cells (PBMCs) (Cron et al., 2019; Witwer, 2015). Several studies have analyzed the miRNA profiles of different types of MG and found some miRNA could be potentially used for diagnosing MG, predicting the therapeutic response and even distinguishing the various MG subtypes (Punga and Punga, 2018; Sabre et al., 2020). In this section, we comprehensively review the miRNA profiles specific to different MG subtypes (Table 1).

**TABLE 1:** Differentially Expressed miRNA.

Autoantibody	Sample	Group	miRNA	Reference
AChR	PBMC	MG vs. HC	↑: miR-146a	Bortone et al. (2020)
AChR	Serum	MG vs. HC	↑: miR-150-5p, miR-21-5p	Punga et al. (2015)
AChR	Serum	MG vs. HC	↓: miR-146a	Bortone et al. (2020)
AChR	Thymus	MG vs. HC	↓: miR-146a	Bortone et al. (2020)
AChR	PBMC	EOMG vs. HC	↓: miR-150-5p	Cron et al. (2019)
AChR	PBMC	EOMG vs. HC	↑: miR-612, miR-3654, pre-miR-3651, miR-3651	Barzago et al. (2016)
AChR	Serum	EOMG vs. HC	↓: miR-15b, miR-192, miR-122, miR-20b, miR140-3p, miR-185	Nogales-Gadea et al. (2014)
AChR	Serum	EOMG vs. HC	↓: miR-20b	Chunjie et al. (2015)
AChR	Whole blood	Israeli cohort: MG-IMM Responser vs. MG-IMM non-Responser	↑: miR-323b-3p, miR-485-3p ↓: miR-181-5p, miR-340-3p	Cavalcante et al. (2019)
AChR	Whole blood	Italian cohort: MG-IMM Responser vs. MG-IMM non-Responser	↑: miR-323b-3p, miR-409-3p, miR-485-3p	Cavalcante et al. (2019)
AChR	Serum	LOMG vs. HC	↓: miR-15b, miR-192, miR-122, miR-20b, miR140-3p, miR-885-5p, miR-185	Nogales-Gadea et al. (2014)
AChR	Serum	TAMG vs. HC	↓: miR-15b	Nogales-Gadea et al. (2014)
AChR	Serum	LOMG-OMG vs. LOMG-SGMG	↑: miR-30e-5p, miR-150-5p	Sabre et al. (2019)
AChR	Serum	MG + IMM vs. MG	↓: miR-150-5p	Punga et al. (2015)
AChR	Serum exosome	MG + RTX vs. MG	↓: miR-150-5p	Zhong et al. (2020)
AChR	Serum	OMG vs. SGMG	↑: miR-30e-5p	Sabre et al. (2019)
AChR/MuSK	Serum	Femal MG vs. femal HC	↑: miR-21-5p	Fiorillo et al. (2020)
AChR/MuSK	Serum	Femal MG vs. male MG	↑: miR-21-5p	Fiorillo et al. (2020)
MuSK	PBMC	MG vs. HC	↓: miR-340-5p, miR-106b-5p, miR-27a-3p, miR-15a-3p	Tan et al. (2021)
MuSK	Serum	MG vs. HC	↑: miR-151a-3p, let-7a-5p, let-7f-5p, miR-423-5p	Punga et al. (2016)
MuSK	Serum exosome	MG + RTX vs. MG	↓: miR-151a-3p	Zhou et al. (2021)
No specific	Serum	MG vs. HC	↑: miR-30e-5p, miR-150-5p	Beretta et al. (2022)
No specific	Serum exosome	OMG vs. HC	↓: miR-4712-3p, miR-320d, miR-3614-3p	Mu et al. (2024)
No specific	Plasmic small extracellular vesicle	Pediatric MG vs. HC	↓: miR-143-3p	Zhu et al. (2024)
No specific	Serum exosome	OMG vs. GMG	↑: miR-130a-3p, miR-4712-3p, miR-320d, miR-3614-3p, miR-6752-5p	Mu et al. (2024)
No specific	Serum	Ocular LOMG vs. Generalized LOMG	↓: miR-150-5p, miR-21-5p	Sabre et al. (2018)

AChR, acetylcholine receptor antibody; MuSK, muscle-specific tyrosine kinase antibody; PBMC, peripheral blood mononuclear cell; MG, Myasthenia Gravis; HC, healthy control; TAMG, thymoma associated MG; EOMG, early onset MG; LOMG, late onset MG; OMG, ocular MG; GMG, generalized MG; SGMG, secondary GMG; RTX, rituximab; IMM, immunosuppressive therapy. ↑ indicates the upregulation of miRNA and ↓ indicates the downregulation of miRNA.

#### 2.1.1 General miRNA profile in MG

Several miRNAs are dysregulated in MG patients (Huang et al., 2023; Wang and Zhang, 2020). In this section, we primarily review miRNA dysregulated generally in MG. The aberrant miRNAs in each MG subtype will be discussed later.

Among these miRNA, miR-150 and miR-21 are most researched in MG. Compared with both healthy individuals and patients with other autoimmune diseases such as Addison’s disease and Crohn’s disease, these two miRNAs are significantly upregulated in serum of MG patients (Punga et al., 2015). In addition, miR-21 is much higher in serum of females. This may, to some extent, explain why MG is more prevalent in women (Fiorillo et al., 2020). Besides that, miR-30e is also found to be unregulated in serum of MG patients, and high level of miR-30e may indicate the progression of MG (Sabre et al., 2019). Meanwhile, another study also showed that MG patient with elevated miR-30e-5p exhibited a higher rate of MG progression (Beretta et al., 2022).

Furthermore, miRNA can also be affected under the influence of treatment and some miRNAs can even predict the treatment response in MG patients. Serum miR-150 and miR-21 decreases after immunosuppressive treatment (Sabre et al., 2018). Low dose rituximab can also decrease serum miR-150-5p, along with improved symptoms and reduced CD19^+^ and CD27^+^ B cells (Zhong et al., 2020). Moreover, international research conducted in Italy and Israel indicated that miR-323b-3p and miR-485-3p in whole blood could predict the response to immunosuppressive agents in MG (Cavalcante et al., 2019). The miR-323b-3p and miR-485-3p decreased in MG patients with poor responses to immunosuppressive treatment. Recently, a researcher explored the dysregulated small extracellular vesicle microRNAs in the plasma of pediatric MG patients. Through RNA sequencing technology, researches identified 24 dysregulated small extracellular vesicle microRNAs (16 downregulated and 9 upregulated). Subsequent validation revealed that plasma miR-143-3p is downregulated in seronegative pediatric MG and can be used for diagnosing pediatric MG (Zhu et al., 2024).

#### 2.1.2 miRNA profile in EOMG

EOMG is one of the most common types of MG. EMOG frequently occurs in younger females and is more likely to accompany thymic hyperplasia (Cron et al., 2018; Fang et al., 2015). It has been indicated that ectopic GC formed in hyperplastic thymus plays crucial role in EOMG (Cron et al., 2018).

Many studies identified several dysregulated miRNAs in EOMG patient, including the upregulation of miR-612, miR-3654, pre-miR-3651, and miR-3651 in PBMCs, miR-150 and miR-21 in serum, and the downregulation of miR-122, miR-140-3p, miR-185, miR-192, miR-20, miR-15b and miR-27a in serum (Nogales-Gadea et al., 2014; Barzago et al., 2016; Punga et al., 2014). Some of these dysregulated miRNAs could be affected by the treatment of MG. In peripheral blood, the abnormally upregulated serum miR-150 could dropped quickly after thymectomy and the abnormally decreased serum miR-20 increased after administration of immunosuppressive agents (Punga et al., 2015). Notably, the level of serum miR-20 negatively correlates with the severity of MG (Chunjie et al., 2015). However, despite the upregulation of thymic miR-150 in MG patients, there is no decline in thymic miR-150 after immunosuppressive therapy (Cron et al., 2019).

It should be noted that the changes of dysregulated miRNA could differ considerably in peripheral blood and thymus. Research explored the role of miR-146a in peripheral blood and hyperplastic thymus in MG patients (Bortone et al., 2020). The results indicated that thymic miR-146a decreased in MG patients without receiving corticosteroids and its level increased to normal levels after treated with corticosteroids. Because corticosteroids have been shown to reduce the number and size of ectopic GC in the hyperplastic thymus of MG patients (Berrih-Aknin, 2016), researchers further explored the relationship between miR-146a and GC in thymus. They found that miR-146a in GC-surrounding tissue rather than in GC was remarkably downregulated in MG patients without receiving corticosteroids. However, the miR-146a in both GC and GC-surrounding tissue is upregulated after treated with corticosteroids. These findings may indicate that corticosteroids could induce the upregulation of miR-146a in MG patients. However, in peripheral blood of corticosteroids-naïve patients, miR-146a was downregulated in serum but upregulated in PBMC. The miR-146a in PBMC then decreased to normal after the administration of corticosteroids. These findings indicated that, in peripheral blood, decreased serum miR-146a might be due to a defect in PBMC secretion of miR-146a and corticosteroids could improve this secretion defect.

#### 2.1.3 miRNA profile in LOMG

LOMG is more commonly seen in elderly patients and more likely to accompany thymoma (Fan et al., 2019). Due to its lower incidence compared with EOMG, there is less research focusing on LOMG.

It has been shown that several miRNAs are dysregulated in the serum of LOMG patients, including the upregulation of miR-106, miR-30e, miR-223, miR-140, and miR-19b and downregulation of miR-122, miR-140, miR-185, miR-192, miR-20b, and miR-15b (Sabre et al., 2018; Nogales-Gadea et al., 2014). Some of these could be affected by immunosuppressive therapy and correlate with the severity of MG. In a study comparing the changes in miRNA over 2 years in LOMG patients, the level of serum miR-150, miR-21 and miR-30e decreased along with an improvement in muscle strength after 1 year follow-up (Sabre et al., 2018). Among a subgroup of LOMG patients, serum miR-150 and miR-21 were found to be lower in the ocular group than in the generalized group. In the generalized group, serum miR-30e, miR-21 and miR-19b was decreased after immunosuppressive therapy. In ocular LOMG, only serum miR-140 was decreased after 1 year follow-up. Moreover, serum miR-150, miR-21, and miR-30e were positively correlated with the Myasthenia Gravis Composite (MGC) score in the subgroup of generalized MG. However, these three miRNAs might not have a potential as biomarkers indicating the severity of LOMG, as all the correlation coefficients are less than 0.3.

#### 2.1.4 miRNA profile in MuSK^+^MG

Despite being a major subtype of MG, MuSK^+^ MG only occurs in <5% of MG patients (Gilhus and Verschuuren, 2015). It is generally assumed that pathogenesis of MuSK^+^ MG is largely unrelated to the thymus. Therefore, clinical guideline recommends using immunosuppressants and rituximab, rather than thymectomy, for treating MuSK^+^ MG without thymic tumors (El-Salem et al., 2014). Consequently, investigators are more interested in the changes of circulating miRNAs in peripheral blood of MuSK^+^ MG patients.

It has been shown that serum miR-151a, let-7a, let-7f, and miR-423, plasma miR-210 and miR-324, as well as PBMC miR-340, miR-106b, and miR-27a are upregulated in MuSK^+^ MG patients (Punga et al., 2016; Tan et al., 2021). Among these, exosomal miR-151a in peripheral blood levels reduced quickly after being treated with immunosuppressants (Zhou et al., 2021). However, due to the limited studies on MuSK^+^ MG, no miRNAs have been found to be associated with the severity of MuSK^+^ MG currently. Therefore, identifying miRNAs that can predict the therapeutic response of MuSK^+^ MG still requires more extensive and in-depth research in the future.

#### 2.1.5 miRNA profile in OMG and GMG

MG can be classified based on the extent of involved muscle, including OMG, in which symptoms are limited to the eye muscles, and GMG, which involves muscles throughout the body, including the limbs and trunk. In the progression of OMG, about 80% of patients with OMG progress to secondary GMG (sGMG) (Wong et al., 2014). During this process, the miRNA profile also changes.

It has been revealed that exosomal miR-106a, miR-4712, miR-320d, and miR-3614-3p is decreased in OMG in contrast to healthy controls (Mu et al., 2024). These four miRNAs are further reduced in GMG compared with the OMG and shows promise as a potential biomarker for diagnosing OMG and GMG (AUC > 0.75), despite limited sample size. Another study with a larger sample size demonstrated the serum miR-30e is higher in sGMG compared with OMG, showing promising potential for distinguishing between OMG and sGMG (AUC > 0.9) (Sabre et al., 2019). While researches on OMG and GMG remains limited, these studies have already identified several miRNAs that can be used for diagnosing OMG, as well as for distinguishing between GMG and OMG. The diagnostic efficacy of these miRNAs still requires validation through prospective studies.

### 2.2 The impact of aberrant miRNA expression on immune cells

**FIGURE 1:**
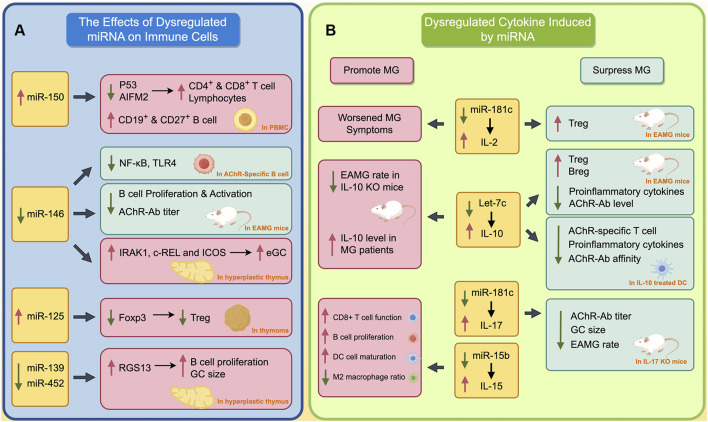
The Impact of Dysregulated miRNAs on Immune Cells and Cytokines in MG. **(A)** The effects of dysregulated miRNA on immune cells; **(B)** Dysregulated cytokines induced by miRNA. The red boxes indicate the processes that promote the progression of MG, while green boxes indicate processes that inhibit the progression of MG. A red upward arrow indicates that this process is enhanced, while a green downward arrow indicates that this process is diminished.

Dysregulated miRNAs play an important role in the pathogenesis of MG, influencing various pathological processes (Berrih-Aknin and Le Panse, 2014). These miRNAs impact immune cell development within the thymus and PBMCs. Additionally, they modulate cytokines and transcription factors, potentially contributing to the thymic alterations observed in MG. These alterations encompass impaired negative selection, ectopic GCs, and angiogenesis (Berrih-Aknin and Le Panse, 2014). In this section, we delve into the specific effects of miRNAs on both the thymus and PBMCs (Figure 1A, by Figdraw).

#### 2.2.1 The effects of miR-150 on immune cells

miR-150 is the most studied miRNA associated with MG. Normally, miRNA could regulate the expression of multiple transcript factors, such as Batch2 and EGR2, thereby participating in the differentiation of immune cells (Huang et al., 2023; Cron et al., 2020; Cron et al., 2019). It has been found that miR-150 is involved in the differentiation and maturation of T cells, B cells and NK cells (Huang et al., 2015). Due to its significant role in the immune system, the dysregulated regulation of miR-150 is implicated in the development of autoimmune disorders such as MG and multiple sclerosis.

In hyperplastic thymus of MG patients, miR-150 is significantly upregulated and primarily located in the mantle zone of ectopic germinal centers (Cron et al., 2019). In addition, the level of miR-150 was positively correlated with CD19, which is a marker of B cells. In peripheral blood, the level of miR-150 in the PBMCs of MG patients was lower than in healthy controls, while serum miR-150 level was significantly higher than in healthy individual. This is thought to occur due to the release of miR-150 from PBMC into serum under abnormal pathological conditions in peripheral blood of MG patients. Further studies found the inhibition of miR-150 could upregulate the expression of p53 and AIFM2, and thus induce the apoptosis of lymphocytes, CD4^+^ and CD8^+^ T cells, which complies with the previously mentioned function of miR-150 in inducing immune cell maturation. Another study found the level of exosomal miR-150 is positively correlated with the level of CD19+B and CD27+B cells (Zhong et al., 2020). This may be attributed to the ability of miR-150 to regulate its target gene c-Myb, thereby influencing the differentiation of B cells into CD19 and CD27 B cells (Xiao et al., 2016).

#### 2.2.2 The effects of miR-146 on immune cells

miR-146 has been identified as a negative regulator of the NF-κB signaling pathway in human immunity (Liu et al., 2015). The activation of miR-146a reduces pro-inflammatory cytokines by inhibiting IRAK1 and TRAF6. In miR-146a deficient mice, NF-κB mediated transcription is abnormally activated (Zhao et al., 2011).

In MG, miR-146a levels are significantly upregulated in PBMC, but downregulated in the hyperplastic thymus (Bortone et al., 2020). This different changing pattern of miR-146a in peripheral and central collectively promotes the progression of MG through different mechanisms. In the mice mode of experimental autoimmune myasthenia gravis (EAMG), inhibition of miR-146 remarkably reduces the expression of TLR4 and NF-κB on AchR specific B cells (Lu et al., 2013). This finding contrasts with previous studies showing the inhibitory effect of miR-146 on the NF-κB signaling pathway, and is assumed to result from a functional deficiency of miR-146a in AchR-specific B cells in MG (Zhao et al., 2011). Another study showed that the inhibition of miR-146 could relieve the myasthenic symptoms of EAMG mice, reduce the production of AChR-Ab, and suppress the proliferation and activation of B cells (Zhang et al., 2015). These two studies demonstrate that, in peripheral blood, miR-146 promotes MG by inducing activation and proliferation of B cell. However, in hyperplastic thymus, downregulated miR-146 may promote the formation of ectopic GC by inducing the expression of IRAK1, c-REL, and ICOS, thereby contributing to the development of MG (Bortone et al., 2020).

#### 2.2.3 The effects of other miRNAs on immune cells

As mentioned earlier, dysfunction of immune cells in both thymus and peripheral blood is a characteristic of MG. Apart from miR-150 and miR-146, other miRNAs are also involved in these complicated processes. In TAMG, the abnormally increased miR-125a could inhibit the expression of Foxp3, and thus reduce the Treg cell in thymoma (Li et al., 2016). The defective Treg results in the inability to suppress excessive inflammation in MG, therefore promoting progression of the disease. Similarly, in the hyperplastic thymus of MG patients, the aberrant downregulation of miR-139-5p and miR-452-5p can increase the expression of RGS13, thereby promoting B cell differentiation and increasing the size of ectopic GC (Sengupta et al., 2018).

### 2.3 Dysregulated cytokine induced by miRNAs

The abnormally activated inflammatory responses in autoimmune reactions is a crucial pathological process of MG (Huda, 2023). During this process, several miRNAs lead to the aberrant secretion and dysfunction of various cytokines, thus resulting in a hyperactive and inflammatory environment (Berrih-Aknin and Le Panse, 2014). In this section, we will summarize the abnormal cytokines in this process, as well as the miRNAs regulating these cytokines (Figure 1B, by Figdraw).

#### 2.3.1 IL-2 in MG

As IL-2 plays an important role in negative selection in the thymus during T cell maturation and maintaining the proper function of Treg in peripheral, dysregulation of IL-2 can be found in several autoimmune diseases such as SLE, RA, and of course, MG (Li et al., 2022; Kolios et al., 2021). IL-2 plays a complex and diverse regulatory role in MG. It has been found that the serum IL-2 level is significantly upregulated in MG patients, but this increase is not positively correlated with the severity of the disease (Huan et al., 2022). In an EAMG mice model, IL-2 secreted by NKT could expand the Treg cells, and thus relieve the myasthenia symptom of mice (Liu et al., 2005). Furthermore, IL-2/IL-2 mAb complex is found to expand the Treg cells in EAMG mice, and therefore suppress the autoreactive T and B cells, thereby relieving the myasthenia symptom of EAMG mice (Liu et al., 2010). However, another study also found that MG patients whose PBMC have strong capacity to secrete more IL-2 after stimulating, often exhibit more severe generalized myasthenia symptoms (Utsugisawa et al., 2003). This result indicates that IL-2 might promote the development of MG. Previous study showed miR-181c could repress the expression of IL-2 by targeting its 3′UTR. Thus, the downregulation of miR-181c in PBMC of MG patients could induce the expression of IL-2 (Utsugisawa et al., 2003; Zhang et al., 2016).

#### 2.3.2 IL-10 in MG

IL-10 plays a complicated role in progression of MG. Early studies found that serum IL-10 levels are elevated in MG patients, and IL-10 knockout mice have a lower incidence of MG when inducing EAMG models (Poussin et al., 2000; Uzawa et al., 2014). Therefore, IL-10 is thought to promote MG. However, in recent years, studies have found that IL-10 may suppress the development of MG. B cell-derived IL-10 is significantly downregulated in MG patients (Yilmaz et al., 2015). A previous study shows that IL-10-competent B cells increase the proportion of immunoregulatory Treg and Breg cells, reduce the levels of proinflammatory cytokines and AChR-Ab, and therefore alleviate the myasthenic symptom of EAMG mice (Sheng et al., 2015). Furthermore, IL-10-treated DC could reduce the AChR-specific T cell proliferation, level of pro-inflammatory cytokines, and affinity of AChR-Ab (Duan et al., 2004). IL-10 can be regulated by let-7c. The let-7c could bind the 3′UTR of IL-10 and inhibit the expression of IL-10 (Jiang et al., 2012). In MG patients, the let-7c is significantly downregulated, which may contribute to the upregulation of serum IL-10 in MG (Jiang et al., 2012).

#### 2.3.3 IL-17 in MG

IL-17 is thought to be a proinflammatory cytokines. A previous study found that serum IL-17 is unregulated in both AChR^+^ and MuSK^+^ MG (Roche et al., 2011; Li et al., 2020). Normally, Treg cells could suppress abnormally overactivated inflammation. However, in the thymus of MG patients, the IL-17 gene family is found to be upregulated, indicating that Treg cells are functionally deficient in MG (Gradolatto et al., 2014; Gradolatto et al., 2012; Villegas et al., 2018). Moreover, it is more difficult to induce EAMG in IL-17 knockout mice compared with wild-type mice (Schaffert et al., 2015). Even in EAMG mode, IL-17 knockout mice exhibit lower levels of myasthenia symptoms and AChR-Ab. The germinal center area in the spleen of IL-17 knockout mice is also smaller (Schaffert et al., 2015; Aguilo-Seara et al., 2017). The aforementioned studies all demonstrate that IL-17 plays a promotive role in the progression of MG. Previous studies have found that miR-15a, miR-320a, and miR-181c are negatively correlated with IL-17 (Zhang et al., 2016; Liu et al., 2016; Cheng et al., 2013). Among these miRNAs, miR-181c could inhibit the expression of IL-17 by targeting its 3′UTR (Zhang et al., 2016). This may indicate that the decrease of miR-181c contributes to the increase in IL-17 to a certain extent in MG patients.

Despite the promotive role that IL-17 plays in MG, it is reported that inhibition of IL-17 by monoclonal antibody could induce MuSK^+^ MG (Papi et al., 2023). This finding suggests that the role of IL-17 in MG may be complex, and thus the use of IL-17 monoclonal antibodies for MG therapy warrants careful investigation.

#### 2.3.4 Other chemokines in MG

The complex pathogenesis of MG is also accompanied by abnormalities in various cytokines, including TNF-α, IL-15, and IL-23. As one of the most well-known pro-inflammatory cytokines, TNF-α is found to be upregulated in both thymus and serum of MG patients (Berrih-Aknin and Le Panse, 2014). Previous studies have indicated that miR-146a may be involved in regulating TNF-α (Nahid et al., 2009). TNF-α is significantly upregulated in miR-146a knockout mice, whereas the mimic of miR-146a was found to inhibit the expression of TNF-α (Yan et al., 2021).

IL-15 is another proinflammatory cytokine that is involved in the regulation of multiple immune cells including enhancing the cytotoxic function of CD8^+^ T cells, inducing B cell proliferation and immunoglobulin secretion, promoting dendritic cell maturation, and reducing the population of M2 macrophages (Zhang et al., 2021). In MG patients, the serum IL-15 level is significantly upregulated and might correlate with the severity of MG (Li et al., 2019). MiR-15b could inhibit the expression of IL-15 by targeting its 3′UTR (Shi et al., 2015). Thus, the reduction of miR-15b could promote the level of IL-15 in MG.

The pro-inflammatory effect of IL-23 is largely associated with IL-17. In MG, the abnormal thymic epithelial cells (TECs) could secrete IL-23, leading to the activation of Th17 cells and subsequently promoting the release of another pro-inflammatory cytokine IL-17 (Villegas et al., 2019). Though this IL23/Th17 axis, the ectopic GCs can be maintained and lead to persistent inflammatory activation in MG (Villegas et al., 2019). Based on the promotive role that IL-23 plays in MG progression, researchers found that blocking IL-23 can alleviate myasthenia symptoms effectively in EAMG mice (Villegas et al., 2023).

Moreover, miRNA is also involved in the metabolism of drug for MG treatment. CYP3A5 encodes a member of the cytochrome P450 superfamily, playing a crucial role in the metabolism of tacrolimus. A study showed that miR-500a could directly inhibit the expression of CYP3A5, and further experiments demonstrated that this miRNA has the potential to maintain the contraction of tacrolimus *in vitro* (Meng et al., 2020).

## 3 The role of lncRNA and circRNA in the diagnosing and pathogenesis of MG

LncRNAs are non-coding RNAs that are more than 200 nucleotides long. circRNAs are circular non-coding RNAs formed by back-splicing of coding and non-coding transcripts (Mattick et al., 2023). Although there is less research on lncRNA and circRNA compared with miRNA in MG, they still play roles in the pathological processes of MG. By high-throughput sequencing, multiple studies have found that lncRNA and circRNA from different samples may play various roles in MG patients. In PBMC, the differentially expressed lncRNAs (DElncRNA) are mainly related to phosphoric ester hydrolase activity and phosphatase activity (Ke et al., 2022). In myotubes treated with AChR-Ab, the DElncRNA is mainly enriched in cellular homeostasis (Hong et al., 2020). In thymoma of TAMG patients, the DElncRNAs were mainly enriched in herpes simplex virus 1 infection, transcription coregulator activity and actin binding (Zhuang et al., 2021). Among these dysregulating lncRNAs and circRNAs, some of them such as IFNG-AS1 in PBMC and circRNA5333-4 in whole blood, are correlated with the severity and AChR-Ab (Lv et al., 2021; Luo et al., 2017).

**FIGURE 2:**
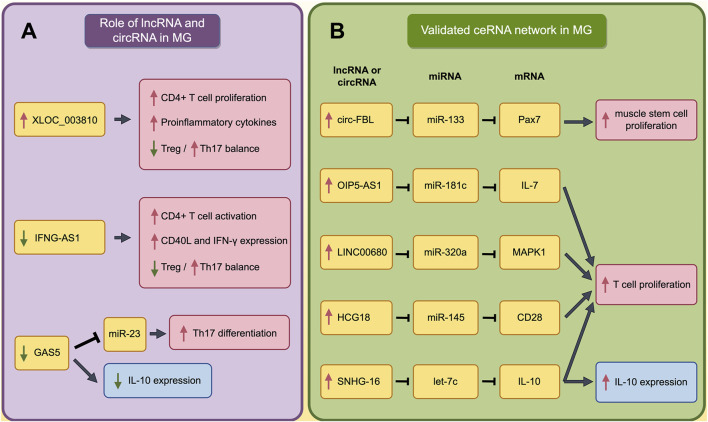
The Impact of Dysregulated lncRNA and validated ceRNA network on Immune Cells and Cytokines in MG. **(A)** The role of dysregulated lncRNAs and circRNAs in pathogenesis of MG; **(B)** The validated ceRNA network in pathogenesis of MG. The red boxes indicate the processes that promote the progression of MG, while the blue boxes indicate that these processes play complex roles in the pathogenesis of MG and cannot be simply categorized as promoting or inhibiting MG. A red upward arrow indicates that this process is enhanced, while a green downward arrow indicates that this process is diminished.

Within MG pathogenesis, lncRNAs have been found to be primarily involved in the dysregulation of immune cells and the abnormal secretion of cytokines. lncRNA XLOC_003810 is highly expressed in the thymus of MG patients. The upregulated XLOC_003810 can increase the proportion of CD4^+^T cells, and elevate the levels of pro-inflammatory cytokines, including IFN-γ, TNF-α and IL-1β (Hu et al., 2020). Moreover, XLOC_003810 was also found to disrupt the Treg/Th17 balance by reducing Treg cells and increasing Th17 cells (Niu et al., 2020). Through the aforementioned mechanisms, XLOC_003810 may contribute to abnormal immune activation in MG. Meanwhile, lncRNA GAS5 could upregulate the IL-10 in GMG patients and suppress the Th17 differentiation by targeting miR-23a. Consequently, the abnormal reduced GAS5 in MG promotes the Th17 differentiation, thereby advancing the progression of MG (Peng and Huang, 2022; Xu and Ouyang, 2022). In addition, lncRNA IFNG-AS1 is downregulated in PBMC of MG and IFNG-AS1 level is negatively correlated with MG severity. Further study has found the IFNG-AS1 could regulate the Treg/Th17 balance by increasing the Treg and decreasing the Th17. Furthermore, IFNG-AS1 can upregulate the expression of CD40L and IFN in CD4^+^ T cells and activate CD4^+^ T cells in an HLA-DOB- and HLA-DRB1-dependent manner (Luo et al., 2017). The roles of dysregulated lncRNAs in MG are summarized in Figure 2A (by Figdraw) and Table 2.

**TABLE 2:** Dysregulated lncRNA in MG.

LncRNA	Changes in MG	Function	References
XLOC_003810	Upregulated	Promoting T cell proliferation and pro-inflammatory cytokines secretion, disrupting the Treg/Th17 balance by reducing Treg cells and increasing Th17 cells	Hu et al. (2020), Niu et al. (2020)
IFNG-AS1	Downregulated	Regulating the Treg/Th17 balance by increasing the Treg and decreasing the Th17, activating CD4^+^ T cells, and upregulating CD40L and IFN expression	Luo et al. (2017)
GAS5	Downregulated	Suppressing the Th17 differentiation and upregulating the expression of IL-10	Peng and Huang (2022), Xu and Ouyang (2022)

## 4 Non-coding RNA networks involved in the MG progression

By competitively binding to miRNAs, lncRNAs and circRNAs can positively regulate mRNA expression through the competing endogenous RNA (ceRNA) network (Thomson and Dinger, 2016). The ceRNA hypothesis has been shown to be involved in many physiological and pathological processes, such as immune response, inflammation response, tumor development and, of course, MG (Zhang et al., 2020; Wang et al., 2020). Moreover, researchers also introduced several networks such as TF-lncRNA-target gene network and the lncRNA‐SNP‐mRNA‐pathway in MG (Lu et al., 2021; Wang et al., 2021). We would then review the research, focusing on the noncoding RNA-based network to help fully understand the pathological process of MG.

### 4.1 ceRNA networks in MG

In autoimmune diseases, dysregulated long non-coding RNAs regulate mRNA expression through ceRNA mechanisms, thereby contributing to the progression of autoimmune diseases such as rheumatoid arthritis (RA), dermatomyositis, and systemic lupus erythematosus (SLE) (Wang et al., 2019; Huang et al., 2022; Li et al., 2017). ceRNA networks can be predicted through bioinformatics methods and validated through further experiments (Wang et al., 2020; Wang et al., 2022). In studies involving ceRNA in MG, we found that validation studies mainly focus on the dysregulated ceRNA axis in peripheral tissue of MG patients. In contrast, bioinformatics-predicted ceRNA networks also highlight potential dysregulated ceRNA networks in the thymus and those involving methylated genes.

#### 4.1.1 Validation studies of ceRNA in MG

It has been found the muscle stem cells (as known as satellite cells, SC) proliferated actively in MG patient (Attia et al., 2017). As a marker for SCs, Pax7 expression in MG patients is regulated by ceRNA networks. Researchers have found that the upregulated circ-FBL in MG patients can increase Pax7 levels by competitively binding to miR-133 (Lai et al., 2021). Moreover, other studies have found that several lncRNAs could promote the proliferation of T cells through ceRNA network. LINC00680 is highly expressed in the PBMCs of MG patients, and this lncRNA can upregulate MAPK1 levels by targeting miR-320a (Liu et al., 2022). Similarly, HCG18, which is upregulated in MG patients, can increase CD28 expression by targeting miR-145, and OIP5-AS1 can enhance IL-7 expression by targeting miR-181c. Besides, upregulated SNHG16 promote the expression of IL-10 by sponging let-7c (Wang et al., 2020; Wang et al., 2022; Li et al., 2021). Through these mechanisms, all these four lncRNAs could promote T cell proliferation and contribute to the progression of MG. These processes are summarized in Figure 2B (by Figdraw) and Table 3.

**TABLE 3:** Validated ceRNA Network in MG.

LncRNA/circRNA	miRNA	mRNA	Function	Reference
circ-FBL	miR-133	Pax7	Muscle stem cells proliferation	Lai et al. (2021)
OIP5-AS1	miR-181c	IL-7	Promoting T cell proliferation	Wang et al. (2022b)
LINC00680	miR-320a	MAPK1	Promoting T cell proliferation	Liu et al. (2022)
HCG18	miR-145	CD28	Promoting T cell proliferation	Li et al. (2021)
SNHG16	let-7c-5p	IL-10	Promoting IL-10 expression and promoting T cell proliferation	Wang et al. (2020)

#### 4.1.2 Predictive studies of ceRNA in MG based on bioinformatics analysis

We have previously predicted a ceRNA in thymoma of TAMG by using the RNA-seq data from the TCGA-THYM project (Wang et al., 2022). In the predicted ceRNA network, we further demonstrated that the changes of each component in the LINC00452/miR-204/CHST4 axis comply with the principles of the ceRNA mechanism, and speculate that Treg cells may be regulated by this axis. Another study established two different ceRNA networks based on the methylation level of mRNA and suggested that LINC00173 plays a significant role in the ceRNA network composed of hypomethylated genes (Xu et al., 2021). Additionally, a study predicted a ceRNA network based on dysregulated exosomal lncRNAs (Lu et al., 2021). Researchers found that molecules previously considered to play important roles in MG, such as miR-181c, miR-125, CD28, and CD40, were also arrested in this predicted ceRNA network. This finding provides further evidence that these molecules may be key players in the development of MG.

### 4.2 Other networks in MG

ceRNA is the most studied and recognized noncoding RNA regulatory mechanism, but some researchers have also explored other modes of networks involving noncoding RNAs in MG. Luo and colleagues constructed the transcription factor-lncRNA-target gene networks in TAMG (Luo et al., 2015). In this network, the transcription factor was thought to regulate gene expression by regulating the lncRNA. This three-element network has mechanisms that are somewhat similar to the ceRNA network. Other research constructed a miRNA-regulated drug-pathway network to predict how the new drug can be for to MG treatment (Cao et al., 2017). Researchers find the alemtuzumab, bevacizumab and efalizumab might have potential to treat MG. However, further validation studies are lacking to support the researchers’ hypotheses.

## 5 Conclusion and prospects

Currently, detecting noncoding RNA is still not a routine test for MG diagnosing or for treatment response predicting. Although some miRNA showed encouraging potential as a biomarker, the noncoding RNA, especially miRNA, varies from different MG subtypes and different samples (Huang et al., 2023; Sabre et al., 2020). Some miRNA such as miR-146a, showed completely different variation in serum and PBMC before and after treatment (Bortone et al., 2020). Therefore, the specific characteristics of MG such as thymus lesion, autoantibody type and treatment should be carefully concerned when choosing the miRNA as a biomarker in future prospective studies and MG diagnosing.

In addition, although numerous studies have explored the impact of non-coding RNAs and their regulatory networks on the development and progression of MG, RNA processes such as RNA editing and RNA methylation—known to play significant roles in other autoimmune diseases like RA and SLE—are scarcely studied in MG. Previous research has found that adenosine-to-inosine (A-to-I) editing levels are significantly elevated in patients with RA, SLE, and Sjögren’s syndrome (Vlachogiannis et al., 2020; Roth et al., 2018; Wang et al., 2023). Moreover, A-to-I levels significantly decreased in responding RA patients after treatment, indicating that A-to-I editing may play an important role in these autoimmune diseases (Vlachogiannis et al., 2020). However, research on the role of A-to-I editing in MG is currently lacking. In the field of RNA methylation, researchers have found that the levels of m^6^A RNA methylation in PBMCs and m^5^C RNA methylation in CD4^+^ T cells are significantly reduced in SLE patients (Guo et al., 2020; Wu et al., 2022). In MG, researchers have found that patients with different m^6^A RNA methylation patterns might be associated with varying immune cell infiltration characteristics (Li et al., 2023). But it is regrettable that this study did not measure the m^6^A RNA methylation levels in MG patients. Therefore, the role of RNA processes in the progression of MG remains to be further explored.

The disturbance of immune homeostasis and abnormally activated inflammation are very important pathological processes in MG (Gilhus, 2016; Berrih-Aknin and Le Panse, 2014; El-Salem et al., 2014). The evidence reviewed above, showing that noncoding RNA can widely influence the both processes by regulating the activation of immune cell, impairing the immunoregulatory functions of Treg and inducing the release of proinflammatory factors (Lu et al., 2013; Li et al., 2016; Zhang et al., 2016). However, the role of noncoding RNA in mechanism behind the TAMG as well as MuSK^+^ MG is less explored. Further studies are expected to uncover such mechanism for fully understanding the pathologic process of MG.

## References

[B1] Aguilo-Seara G. Xie Y. Sheehan J. Kusner L. L. Kaminski H. J. (2017). Ablation of IL-17 expression moderates experimental autoimmune myasthenia gravis disease severity. Cytokine 96, 279–285. 10.1016/j.cyto.2017.05.008 28599246

[B2] Ashby K. M. Hogquist K. A. (2024). A guide to thymic selection of T cells. Nat. Rev. Immunol. 24 (2), 103–117. 10.1038/s41577-023-00911-8 37464188

[B3] Attia M. Maurer M. Robinet M. Le Grand F. Fadel E. Le Panse R. (2017). Muscle satellite cells are functionally impaired in myasthenia gravis: consequences on muscle regeneration. Acta Neuropathol. 134 (6), 869–888. 10.1007/s00401-017-1754-2 28756524

[B4] Barzago C. Lum J. Cavalcante P. Srinivasan K. G. Faggiani E. Camera G. (2016). A novel infection- and inflammation-associated molecular signature in peripheral blood of myasthenia gravis patients. Immunobiology 221 (11), 1227–1236. 10.1016/j.imbio.2016.06.012 27387891

[B5] Beretta F. Huang Y. F. Punga A. R. (2022). Towards personalized medicine in myasthenia gravis: role of circulating microRNAs miR-30e-5p, miR-150-5p and miR-21-5p. Cells 11 (4), 740. 10.3390/cells11040740 35203389 PMC8870722

[B6] Berrih-Aknin S. (2016). Role of the thymus in autoimmune myasthenia gravis. Clin. Exp. Neuroimmunol. 7 (3), 226–237. 10.1111/cen3.12319

[B7] Berrih-Aknin S. Le Panse R. (2014). Myasthenia gravis: a comprehensive review of immune dysregulation and etiological mechanisms. J. Autoimmun. 52, 90–100. 10.1016/j.jaut.2013.12.011 24389034

[B8] Blow M. J. Grocock R. J. van Dongen S. Enright A. J. Dicks E. Futreal P. A. (2006). RNA editing of human microRNAs. Genome Biol. 7 (4), R27. 10.1186/gb-2006-7-4-r27 16594986 PMC1557993

[B9] Bortone F. Scandiffio L. Marcuzzo S. Bonanno S. Frangiamore R. Motta T. (2020). miR-146a in myasthenia gravis thymus bridges innate immunity with autoimmunity and is linked to therapeutic effects of corticosteroids. Front. Immunol. 11, 142. 10.3389/fimmu.2020.00142 32210951 PMC7075812

[B10] Cai Y. Yu X. Hu S. Yu J. (2009). A brief review on the mechanisms of miRNA regulation. Genomics Proteomics Bioinforma. 7 (4), 147–154. 10.1016/S1672-0229(08)60044-3 PMC5054406 20172487

[B11] Cao Y. Lu X. Wang J. Zhang H. Liu Z. Xu S. (2017). Construction of an miRNA-regulated drug-pathway network reveals drug repurposing candidates for myasthenia gravis. Int. J. Mol. Med. 39 (2), 268–278. 10.3892/ijmm.2017.2853 28075449 PMC5358695

[B12] Cavalcante P. Mizrachi T. Barzago C. Scandiffio L. Bortone F. Bonanno S. (2019). MicroRNA signature associated with treatment response in myasthenia gravis: a further step towards precision medicine. Pharmacol. Res. 148, 104388. 10.1016/j.phrs.2019.104388 31401213

[B13] Cheng L. Sharples R. A. Scicluna B. J. Hill A. F. (2014). Exosomes provide a protective and enriched source of miRNA for biomarker profiling compared to intracellular and cell-free blood. J. Extracell. Vesicles 3. 10.3402/jev.v3.23743 PMC3968297 24683445

[B14] Cheng Z. Qiu S. Jiang L. Zhang A. Bao W. Liu P. (2013). MiR-320a is downregulated in patients with myasthenia gravis and modulates inflammatory cytokines production by targeting mitogen-activated protein kinase 1. J. Clin. Immunol. 33 (3), 567–576. 10.1007/s10875-012-9834-5 23196978

[B15] Chunjie N. Huijuan N. Zhao Y. Jianzhao W. Xiaojian Z. (2015). Disease-specific signature of serum miR-20b and its targets IL-8 and IL-25, in myasthenia gravis patients. Eur. Cytokine Netw. 26 (3), 61–66. 10.1684/ecn.2015.0367 26845056

[B16] Cron M. A. Guillochon É. Kusner L. Le Panse R. (2020). Role of miRNAs in normal and myasthenia gravis thymus. Front. Immunol. 11, 1074. 10.3389/fimmu.2020.01074 32587589 PMC7297979

[B17] Cron M. A. Maillard S. Truffault F. Gualeni A. V. Gloghini A. Fadel E. (2019). Causes and consequences of miR-150-5p dysregulation in myasthenia gravis. Front. Immunol. 10, 539. 10.3389/fimmu.2019.00539 30984166 PMC6450174

[B18] Cron M. A. Maillard S. Villegas J. Truffault F. Sudres M. Dragin N. (2018). Thymus involvement in early-onset myasthenia gravis. Ann. N. Y. Acad. Sci. 1412 (1), 137–145. 10.1111/nyas.13519 29125185

[B19] Duan R. S. Adikari S. B. Huang Y. M. Link H. Xiao B. G. (2004). Protective potential of experimental autoimmune myasthenia gravis in Lewis rats by IL-10-modified dendritic cells. Neurobiol. Dis. 16 (2), 461–467. 10.1016/j.nbd.2004.03.017 15193302

[B20] El-Salem K. Yassin A. Al-Hayk K. Yahya S. Al-Shorafat D. Dahbour S. S. (2014). Treatment of MuSK-associated myasthenia gravis. Curr. Treat. Options Neurol. 16 (4), 283. 10.1007/s11940-014-0283-8 24504626

[B21] Fan L. Ma S. Yang Y. Yan Z. Li J. Li Z. (2019). Clinical differences of early and late-onset myasthenia gravis in 985 patients. Neurol. Res. 41 (1), 45–51. 10.1080/01616412.2018.1525121 30311866

[B22] Fang F. Sveinsson O. Thormar G. Granqvist M. Askling J. Lundberg I. E. (2015). The autoimmune spectrum of myasthenia gravis: a Swedish population-based study. J. Intern Med. 277 (5), 594–604. 10.1111/joim.12310 25251578

[B23] Fiorillo A. A. Heier C. R. Huang Y. F. Tully C. B. Punga T. Punga A. R. (2020). Estrogen receptor, inflammatory, and FOXO transcription factors regulate expression of myasthenia gravis-associated circulating microRNAs. Front. Immunol. 11, 151. 10.3389/fimmu.2020.00151 32153563 PMC7046803

[B24] Gilhus N. E. (2016). Myasthenia gravis. N. Engl. J. Med. 375 (26), 2570–2581. 10.1056/NEJMra1602678 28029925

[B25] Gilhus N. E. Verschuuren J. J. (2015). Myasthenia gravis: subgroup classification and therapeutic strategies. Lancet Neurol. 14 (10), 1023–1036. 10.1016/S1474-4422(15)00145-3 26376969

[B26] Gradolatto A. Nazzal D. Foti M. Bismuth J. Truffault F. Le Panse R. (2012). Defects of immunoregulatory mechanisms in myasthenia gravis: role of IL-17. Ann. N. Y. Acad. Sci. 1274, 40–47. 10.1111/j.1749-6632.2012.06791.x 23252896

[B27] Gradolatto A. Nazzal D. Truffault F. Bismuth J. Fadel E. Foti M. (2014). Both Treg cells and Tconv cells are defective in the Myasthenia gravis thymus: roles of IL-17 and TNF-α. J. Autoimmun. 52, 53–63. 10.1016/j.jaut.2013.12.015 24405842

[B28] Gregory R. I. Chendrimada T. P. Cooch N. Shiekhattar R. (2005). Human RISC couples microRNA biogenesis and posttranscriptional gene silencing. Cell 123 (4), 631–640. 10.1016/j.cell.2005.10.022 16271387

[B29] Guo G. Wang H. Shi X. Ye L. Yan K. Chen Z. (2020). Disease activity-associated alteration of mRNA m(5) C methylation in CD4(+) T cells of systemic lupus erythematosus. Front. Cell Dev. Biol. 8, 430. 10.3389/fcell.2020.00430 32582707 PMC7291606

[B30] Hong Y. Liang X. Gilhus N. E. (2020). AChR antibodies show a complex interaction with human skeletal muscle cells in a transcriptomic study. Sci. Rep. 10 (1), 11230. 10.1038/s41598-020-68185-x 32641696 PMC7343820

[B31] Hu B. Niu L. Jiang Z. Xu S. Hu Y. Cao K. (2020). LncRNA XLOC_003810 promotes T cell activation and inhibits PD-1/PD-L1 expression in patients with myasthenia gravis-related thymoma. Scand. J. Immunol. 92 (1), e12886. 10.1111/sji.12886 32243615

[B32] Huan X. Zhao R. Song J. Zhong H. Su M. Yan C. (2022). Increased serum IL-2, IL-4, IL-5 and IL-12p70 levels in AChR subtype generalized myasthenia gravis. BMC Immunol. 23 (1), 26. 10.1186/s12865-022-00501-8 35624411 PMC9145157

[B33] Huang P. Tang L. Zhang L. Ren Y. Peng H. Xiao Y. (2022). Identification of biomarkers associated with CD4(+) T-cell infiltration with gene coexpression network in dermatomyositis. Front. Immunol. 13, 854848. 10.3389/fimmu.2022.854848 35711463 PMC9196312

[B34] Huang X. Zhang Z. Wang Y. Xu M. Du X. Zhang Y. (2023). Circulating miRNAs drive personalized medicine based on subgroup classification in myasthenia gravis patients. Neurol. Sci. 44 (11), 3877–3884. 10.1007/s10072-023-06933-3 37402938

[B35] Huang X. L. Zhang L. Li J. P. Wang Y. J. Duan Y. Wang J. (2015). MicroRNA-150: a potential regulator in pathogens infection and autoimmune diseases. Autoimmunity 48 (8), 503–510. 10.3109/08916934.2015.1072518 26287504

[B36] Huda R. (2023). Inflammation and autoimmune myasthenia gravis. Front. Immunol. 14, 1110499. 10.3389/fimmu.2023.1110499 36793733 PMC9923104

[B37] Jiang L. Cheng Z. Qiu S. Que Z. Bao W. Jiang C. (2012). Altered let-7 expression in Myasthenia gravis and let-7c mediated regulation of IL-10 by directly targeting IL-10 in Jurkat cells. Int. Immunopharmacol. 14 (2), 217–223. 10.1016/j.intimp.2012.07.003 22835429

[B38] Kai K. Dittmar R. L. Sen S. (2018). Secretory microRNAs as biomarkers of cancer. Semin. Cell Dev. Biol. 78, 22–36. 10.1016/j.semcdb.2017.12.011 29258963

[B39] Ke J. Du X. Cui J. Yu L. Li H. (2022). LncRNA and mRNA expression associated with myasthenia gravis in patients with thymoma. Thorac. Cancer 13 (1), 15–23. 10.1111/1759-7714.14201 34773374 PMC8720629

[B40] Kolios A. G. A. Tsokos G. C. Klatzmann D. (2021). Interleukin-2 and regulatory T cells in rheumatic diseases. Nat. Rev. Rheumatol. 17 (12), 749–766. 10.1038/s41584-021-00707-x 34728817

[B41] Lai X. Bi Z. Yang X. Hu R. Wang L. Jin M. (2021). Upregulation of circ-FBL promotes myogenic proliferation in myasthenia gravis by regulation of miR-133/PAX7. Cell Biol. Int. 45 (11), 2287–2293. 10.1002/cbin.11676 34363272 PMC9290729

[B42] Li H. Boulougoura A. Endo Y. Tsokos G. C. (2022). Abnormalities of T cells in systemic lupus erythematosus: new insights in pathogenesis and therapeutic strategies. J. Autoimmun. 132, 102870. 10.1016/j.jaut.2022.102870 35872102

[B43] Li J. Qiu D. Chen Z. Du W. Liu J. Mo X. (2016). Altered expression of miR-125a-5p in thymoma-associated myasthenia gravis and its down-regulation of foxp3 expression in Jurkat cells. Immunol. Lett. 172, 47–55. 10.1016/j.imlet.2016.02.005 26875774

[B44] Li L. J. Zhao W. Tao S. S. Leng R. X. Fan Y. G. Pan H. F. (2017). Competitive endogenous RNA network: potential implication for systemic lupus erythematosus. Expert Opin. Ther. Targets 21 (6), 639–648. 10.1080/14728222.2017.1319938 28406715

[B45] Li S. Liu H. Ruan Z. Guo R. Sun C. Tang Y. (2023). Landscape analysis of m6A modification regulators related biological functions and immune characteristics in myasthenia gravis. J. Transl. Med. 21 (1), 166. 10.1186/s12967-023-03947-5 36864526 PMC9983271

[B46] Li S. Wang X. Wang T. Zhang H. Lu X. Liu L. (2021). Identification of the regulatory role of lncRNA HCG18 in myasthenia gravis by integrated bioinformatics and experimental analyses. J. Transl. Med. 19 (1), 468. 10.1186/s12967-021-03138-0 34794447 PMC8600732

[B47] Li Y. Guptill J. T. Russo M. A. Howard J. F. Massey J. M. Juel V. C. (2020). Imbalance in T follicular helper cells producing IL-17 promotes pro-inflammatory responses in MuSK antibody positive myasthenia gravis. J. Neuroimmunol. 345, 577279. 10.1016/j.jneuroim.2020.577279 32497931 PMC7397478

[B48] Liu L. Zhang H. Lu X. Li L. Wang T. Li S. (2022). LncRNA LINC00680 acts as a competing endogenous RNA and is associated with the severity of myasthennia gravis. Front. Neurol. 13, 833062. 10.3389/fneur.2022.833062 35800083 PMC9253289

[B49] Liu R. La Cava A. Bai X. F. Jee Y. Price M. Campagnolo D. I. (2005). Cooperation of invariant NKT cells and CD4+CD25+ T regulatory cells in the prevention of autoimmune myasthenia. J. Immunol. 175 (12), 7898–7904. 10.4049/jimmunol.175.12.7898 16339525

[B50] Liu R. Liu C. Chen D. Yang W. H. Liu X. Liu C. G. (2015). FOXP3 controls an miR-146/NF-κB negative feedback loop that inhibits apoptosis in breast cancer cells. Cancer Res. 75 (8), 1703–1713. 10.1158/0008-5472.CAN-14-2108 25712342 PMC4706751

[B51] Liu R. Zhou Q. La Cava A. Campagnolo D. I. Van Kaer L. Shi F. D. (2010). Expansion of regulatory T cells via IL-2/anti-IL-2 mAb complexes suppresses experimental myasthenia. Eur. J. Immunol. 40 (6), 1577–1589. 10.1002/eji.200939792 20352624 PMC3600978

[B52] Liu X. F. Wang R. Q. Hu B. Luo M. C. Zeng Q. M. Zhou H. (2016). MiR-15a contributes abnormal immune response in myasthenia gravis by targeting CXCL10. Clin. Immunol. 164, 106–113. 10.1016/j.clim.2015.12.009 26845678

[B53] Li X. Lu F. Li W. Zhao D. Li X. Zhang J. (2019). The correlation of the levels of interleukin-15 with late onset myasthenia gravis. Chin. J. Neurology, 446–451. 10.3760/cma.j.issn.1006-7876.2019.06.002

[B54] Lu J. Yan M. Wang Y. Zhang J. Yang H. Tian F. F. (2013). Altered expression of miR-146a in myasthenia gravis. Neurosci. Lett. 555, 85–90. 10.1016/j.neulet.2013.09.014 24036458

[B55] Lu W. Lu Y. Wang C. F. Chen T. T. (2021). Expression profiling and bioinformatics analysis of exosomal long noncoding RNAs in patients with myasthenia gravis by RNA sequencing. J. Clin. Lab. Anal. 35 (4), e23722. 10.1002/jcla.23722 33543801 PMC8059713

[B56] Luo M. Liu X. Meng H. Xu L. Li Y. Li Z. (2017). IFNA-AS1 regulates CD4(+) T cell activation in myasthenia gravis though HLA-DRB1. Clin. Immunol. 183, 121–131. 10.1016/j.clim.2017.08.008 28822831

[B57] Luo Z. Li Y. Liu X. Luo M. Xu L. Luo Y. (2015). Systems biology of myasthenia gravis, integration of aberrant lncRNA and mRNA expression changes. BMC Med. Genomics 8, 13. 10.1186/s12920-015-0087-z 25889429 PMC4380247

[B58] Lv J. Ren L. Han S. Zhang J. Zhao X. Zhang Y. (2021). Peripheral blood hsa-circRNA5333-4: a novel biomarker for myasthenia gravis. Clin. Immunol. 224, 108676. 10.1016/j.clim.2021.108676 33465495

[B59] Mattick J. S. Amaral P. P. Carninci P. Carpenter S. Chang H. Y. Chen L. L. (2023). Long non-coding RNAs: definitions, functions, challenges and recommendations. Nat. Rev. Mol. Cell Biol. 24 (6), 430–447. 10.1038/s41580-022-00566-8 36596869 PMC10213152

[B60] Meng H. Y. Li X. Jin W. L. Yan C. K. Dong X. H. Xu Q. (2020). Multiple genetic factors affecting the pharmacokinetic and pharmacodynamic processes of tacrolimus in Chinese myasthenia gravis patients. Eur. J. Clin. Pharmacol. 76 (5), 659–671. 10.1007/s00228-019-02803-0 31955224

[B61] Monsul N. T. Patwa H. S. Knorr A. M. Lesser R. L. Goldstein J. M. (2004). The effect of prednisone on the progression from ocular to generalized myasthenia gravis. J. Neurol. Sci. 217 (2), 131–133. 10.1016/j.jns.2003.08.017 14706214

[B62] Mu Y. Huang X. Yang Y. Huang Z. Chen J. Li S. (2024). Study of serum exosome miRNA as a biomarker for early onset adult ouclar myastthenia gravis. Gene 896, 148034. 10.1016/j.gene.2023.148034 38013129

[B63] Nahid M. A. Pauley K. M. Satoh M. Chan E. K. L. (2009). miR-146a is critical for endotoxin-induced tolerance: implication in innate immunity. J. Biol. Chem. 284 (50), 34590–34599. 10.1074/jbc.M109.056317 19840932 PMC2787321

[B64] Niu L. Jiang J. Yin Y. Hu B. (2020). LncRNA XLOC_003810 modulates thymic Th17/Treg balance in myasthenia gravis with thymoma. Clin. Exp. Pharmacol. Physiol. 47 (6), 989–996. 10.1111/1440-1681.13280 32048308

[B65] Nogales-Gadea G. Ramos-Fransi A. Suárez-Calvet X. Navas M. Rojas-García R. Mosquera J. L. (2014). Analysis of serum miRNA profiles of myasthenia gravis patients. PLoS One 9 (3), e91927. 10.1371/journal.pone.0091927 24637658 PMC3956820

[B66] Papi C. Granata G. Galluzzo M. Iorio R. (2023). Myasthenia gravis associated with muscle-specific kinase antibodies in a patient treated with interleukin-17 inhibitor. Neurol. Sci. 44 (3), 1105–1107. 10.1007/s10072-022-06498-7 36357816

[B67] Peng S. Huang Y. (2022). LncRNA GAS5 positively regulates IL-10 expression in patients with generalized myasthenia gravis. Brain Behav. 12 (1), e2457. 10.1002/brb3.2457 34936242 PMC8785628

[B68] Poussin M. A. Goluszko E. Hughes T. K. Duchicella S. I. Christadoss P. (2000). Suppression of experimental autoimmune myasthenia gravis in IL-10 gene-disrupted mice is associated with reduced B cells and serum cytotoxicity on mouse cell line expressing AChR. J. Neuroimmunol. 111 (1-2), 152–160. 10.1016/s0165-5728(00)00385-4 11063833

[B69] Punga A. R. Andersson M. Alimohammadi M. Punga T. (2015). Disease specific signature of circulating miR-150-5p and miR-21-5p in myasthenia gravis patients. J. Neurol. Sci. 356 (1-2), 90–96. 10.1016/j.jns.2015.06.019 26095457

[B70] Punga A. R. Punga T. (2018). Circulating microRNAs as potential biomarkers in myasthenia gravis patients. Ann. N. Y. Acad. Sci. 1412 (1), 33–40. 10.1111/nyas.13510 29125182

[B71] Punga T. Bartoccioni E. Lewandowska M. Damato V. Evoli A. Punga A. R. (2016). Disease specific enrichment of circulating let-7 family microRNA in MuSK+ myasthenia gravis. J. Neuroimmunol. 292, 21–26. 10.1016/j.jneuroim.2016.01.003 26943954

[B72] Punga T. Le Panse R. Andersson M. Truffault F. Berrih-Aknin S. Punga A. R. (2014). Circulating miRNAs in myasthenia gravis: miR-150-5p as a new potential biomarker. Ann. Clin. Transl. Neurol. 1 (1), 49–58. 10.1002/acn3.24 25356381 PMC4207504

[B73] Roche J. C. Capablo J. L. Larrad L. Gervas-Arruga J. Ara J. R. Sánchez A. (2011). Increased serum interleukin-17 levels in patients with myasthenia gravis. Muscle Nerve 44 (2), 278–280. 10.1002/mus.22070 21755509

[B74] Roth S. H. Danan-Gotthold M. Ben-Izhak M. Rechavi G. Cohen C. J. Louzoun Y. (2018). Increased RNA editing may provide a source for autoantigens in systemic lupus erythematosus. Cell Rep. 23 (1), 50–57. 10.1016/j.celrep.2018.03.036 29617672 PMC5905401

[B75] Sabre L. Maddison P. Sadalage G. Ambrose P. A. Punga A. R. (2018). Circulating microRNA miR-21-5p, miR-150-5p and miR-30e-5p correlate with clinical status in late onset myasthenia gravis. J. Neuroimmunol. 321, 164–170. 10.1016/j.jneuroim.2018.05.003 29804819

[B76] Sabre L. Maddison P. Wong S. H. Sadalage G. Ambrose P. A. Plant G. T. (2019). miR-30e-5p as predictor of generalization in ocular myasthenia gravis. Ann. Clin. Transl. Neurol. 6 (2), 243–251. 10.1002/acn3.692 30847357 PMC6389736

[B77] Sabre L. Punga T. Punga A. R. (2020). Circulating miRNAs as potential biomarkers in myasthenia gravis: tools for personalized medicine. Front. Immunol. 11, 213. 10.3389/fimmu.2020.00213 32194544 PMC7065262

[B78] Schaffert H. Pelz A. Saxena A. Losen M. Meisel A. Thiel A. (2015). IL-17-producing CD4(+) T cells contribute to the loss of B-cell tolerance in experimental autoimmune myasthenia gravis. Eur. J. Immunol. 45 (5), 1339–1347. 10.1002/eji.201445064 25676041

[B79] Sengupta M. Wang B. D. Lee N. H. Marx A. Kusner L. L. Kaminski H. J. (2018). MicroRNA and mRNA expression associated with ectopic germinal centers in thymus of myasthenia gravis. PLoS One 13 (10), e0205464. 10.1371/journal.pone.0205464 30308012 PMC6181382

[B80] Sheng J. R. Quan S. Soliven B. (2015). IL-10 derived from CD1dhiCD5⁺ B cells regulates experimental autoimmune myasthenia gravis. J. Neuroimmunol. 289, 130–138. 10.1016/j.jneuroim.2015.10.023 26616882

[B81] Shi L. Liu T. Zhang M. Guo Y. Song C. Song D. (2015). miR-15b is downregulated in myasthenia gravis patients and directly regulates the expression of interleukin-15 (IL-15) in experimental myasthenia gravis mice. Med. Sci. Monit. 21, 1774–1780. 10.12659/MSM.893458 26087886 PMC4485652

[B82] Singh R. P. Massachi I. Manickavel S. Singh S. Rao N. P. Hasan S. (2013). The role of miRNA in inflammation and autoimmunity. Autoimmun. Rev. 12 (12), 1160–1165. 10.1016/j.autrev.2013.07.003 23860189

[B83] Skeie G. O. Aarli J. A. Gilhus N. E. (2006). Titin and ryanodine receptor antibodies in myasthenia gravis. Acta Neurol. Scand. Suppl. 183, 19–23. 10.1111/j.1600-0404.2006.00608.x 16637922

[B84] Sprent J. Kishimoto H. (2002). The thymus and negative selection. Immunol. Rev. 185, 126–135. 10.1034/j.1600-065x.2002.18512.x 12190927

[B85] Szczudlik P. Szyluk B. Lipowska M. Ryniewicz B. Kubiszewska J. Dutkiewicz M. (2014). Antititin antibody in early- and late-onset myasthenia gravis. Acta Neurol. Scand. 130 (4), 229–233. 10.1111/ane.12271 24947881

[B86] Tan Y. Zhu L. Cui L. Guan Y. (2021). Differential expression of miRNA in the peripheral blood mononuclear cells in myasthenia gravis with muscle-specific receptor tyrosine kinase antibodies. Crit. Rev. Eukaryot. Gene Expr. 31 (2), 1–15. 10.1615/CritRevEukaryotGeneExpr.2021037369 34347975

[B87] Thomson D. W. Dinger M. E. (2016). Endogenous microRNA sponges: evidence and controversy. Nat. Rev. Genet. 17 (5), 272–283. 10.1038/nrg.2016.20 27040487

[B88] Utsugisawa K. Nagane Y. Obara D. Kondoh R. Yonezawa H. Tohgi H. (2003). Interleukin-2 production by peripheral blood mononuclear cells from patients with myasthenia gravis. Eur. Neurol. 49 (3), 160–163. 10.1159/000069079 12646760

[B89] Uzawa A. Kawaguchi N. Himuro K. Kanai T. Kuwabara S. (2014). Serum cytokine and chemokine profiles in patients with myasthenia gravis. Clin. Exp. Immunol. 176 (2), 232–237. 10.1111/cei.12272 24666229 PMC3992035

[B90] Villegas J. A. Bayer A. C. Ider K. Bismuth J. Truffault F. Roussin R. (2019). Il-23/Th17 cell pathway: a promising target to alleviate thymic inflammation maintenance in myasthenia gravis. J. Autoimmun. 98, 59–73. 10.1016/j.jaut.2018.11.005 30578016

[B91] Villegas J. A. Van Wassenhove J. Le Panse R. Berrih-Aknin S. Dragin N. (2018). An imbalance between regulatory T cells and T helper 17 cells in acetylcholine receptor-positive myasthenia gravis patients. Ann. N. Y. Acad. Sci. 1413 (1), 154–162. 10.1111/nyas.13591 29405352

[B92] Villegas J. A. Van Wassenhove J. Merrheim J. Matta K. Hamadache S. Flaugère C. (2023). Blocking interleukin-23 ameliorates neuromuscular and thymic defects in myasthenia gravis. J. Neuroinflammation 20 (1), 9. 10.1186/s12974-023-02691-3 36639663 PMC9837970

[B93] Vlachogiannis N. I. Gatsiou A. Silvestris D. A. Stamatelopoulos K. Tektonidou M. G. Gallo A. (2020). Increased adenosine-to-inosine RNA editing in rheumatoid arthritis. J. Autoimmun. 106, 102329. 10.1016/j.jaut.2019.102329 31493964 PMC7479519

[B94] Wang F. Zhang H. Qiu G. Li Z. Wang Y. (2022a). The linc00452/miR-204/CHST4 Axis regulating thymic tregs might Be involved in the progression of thymoma-associated myasthenia gravis. Front. Neurol. 13, 828970. 10.3389/fneur.2022.828970 35432149 PMC9005856

[B95] Wang J. Cao Y. Lu X. Wang X. Kong X. Bo C. (2020). Identification of the regulatory role of lncRNA SNHG16 in myasthenia gravis by constructing a competing endogenous RNA network. Mol. Ther. Nucleic Acids 19, 1123–1133. 10.1016/j.omtn.2020.01.005 32059338 PMC7016163

[B96] Wang J. Yan S. Yang J. Lu H. Xu D. Wang Z. (2019). Non-coding RNAs in rheumatoid arthritis: from bench to bedside. Front. Immunol. 10, 3129. 10.3389/fimmu.2019.03129 32047497 PMC6997467

[B97] Wang L. Zhang L. (2020). Emerging roles of dysregulated MicroRNAs in myasthenia gravis. Front. Neurosci. 14, 507. 10.3389/fnins.2020.00507 32508584 PMC7253668

[B98] Wang T. Xu S. Zhang H. Lu X. Li S. Liu L. (2021). Competitive endogenous RNA network and pathway-based analysis of LncRNA single-nucleotide polymorphism in myasthenia gravis. Sci. Rep. 11 (1), 23920. 10.1038/s41598-021-03357-x 34907261 PMC8671434

[B99] Wang X. Zhang H. Lu X. Li S. Kong X. Liu L. (2022b). LncRNA OIP5-AS1 modulates the proliferation and apoptosis of Jurkat cells by sponging miR-181c-5p to regulate IL-7 expression in myasthenia gravis. PeerJ 10, e13454. 10.7717/peerj.13454 35602889 PMC9121865

[B100] Wang X. Zhu L. Ying S. Liao X. Zheng J. Liu Z. (2023). Increased RNA editing sites revealed as potential novel biomarkers for diagnosis in primary Sjögren’s syndrome. J. Autoimmun. 138, 103035. 10.1016/j.jaut.2023.103035 37216868

[B101] Witwer K. W. (2015). Circulating microRNA biomarker studies: pitfalls and potential solutions. Clin. Chem. 61 (1), 56–63. 10.1373/clinchem.2014.221341 25391989

[B102] Wong S. H. Huda S. Vincent A. Plant G. T. (2014). Ocular myasthenia gravis: controversies and updates. Curr. Neurol. Neurosci. Rep. 14 (1), 421. 10.1007/s11910-013-0421-9 24272275

[B103] Wu J. Deng L. J. Xia Y. R. Leng R. X. Fan Y. G. Pan H. F. (2022). Involvement of N6-methyladenosine modifications of long noncoding RNAs in systemic lupus erythematosus. Mol. Immunol. 143, 77–84. 10.1016/j.molimm.2022.01.006 35051888

[B104] Xiao C. Calado D. P. Galler G. Thai T. H. Patterson H. C. Wang J. (2016). MiR-150 controls B cell differentiation by targeting the transcription factor c-myb. Cell 165 (4), 1027. 10.1016/j.cell.2016.04.056 27153500

[B105] Xu S. Wang T. Lu X. Zhang H. Liu L. Kong X. (2021). Identification of LINC00173 in myasthenia gravis by integration analysis of aberrantly methylated- differentially expressed genes and ceRNA networks. Front. Genet. 12, 726751. 10.3389/fgene.2021.726751 34603387 PMC8481885

[B106] Xu Y. Ouyang Y. (2022). Long non-coding RNA growth arrest specific 5 regulates the T helper 17/regulatory T balance by targeting miR-23a in myasthenia gravis. J. Int. Med. Res. 50 (6), 3000605211053703. 10.1177/03000605211053703 35707849 PMC9208058

[B107] Yan F. Wufuer D. Ding J. Wang J. (2021). MicroRNA miR-146a-5p inhibits the inflammatory response and injury of airway epithelial cells via targeting TNF receptor-associated factor 6. Bioengineered 12 (1), 1916–1926. 10.1080/21655979.2021.1927545 34002665 PMC8806598

[B108] Yilmaz V. Oflazer P. Aysal F. Parman Y. G. Direskeneli H. Deymeer F. (2015). B cells produce less IL-10, IL-6 and TNF-α in myasthenia gravis. Autoimmunity 48 (4), 201–207. 10.3109/08916934.2014.992517 25518708

[B109] Yoganathan K. Stevenson A. Tahir A. Sadler R. Radunovic A. Malek N. (2022). Bedside and laboratory diagnostic testing in myasthenia. J. Neurol. 269 (6), 3372–3384. 10.1007/s00415-022-10986-3 35142871 PMC9119875

[B110] You C. He W. Hang R. Zhang C. Cao X. Guo H. (2019). FIERY1 promotes microRNA accumulation by suppressing rRNA-derived small interfering RNAs in Arabidopsis. Nat. Commun. 10 (1), 4424. 10.1038/s41467-019-12379-z 31562313 PMC6765019

[B111] Zhang B. Pan X. Cobb G. P. Anderson T. A. (2007). microRNAs as oncogenes and tumor suppressors. Dev. Biol. 302 (1), 1–12. 10.1016/j.ydbio.2006.08.028 16989803

[B112] Zhang J. Jia G. Liu Q. Hu J. Yan M. Yang B. (2015). Silencing miR-146a influences B cells and ameliorates experimental autoimmune myasthenia gravis. Immunology 144 (1), 56–67. 10.1111/imm.12347 24962817 PMC4264910

[B113] Zhang K. Zhang L. Mi Y. Tang Y. Ren F. Liu B. (2020). A ceRNA network and a potential regulatory axis in gastric cancer with different degrees of immune cell infiltration. Cancer Sci. 111 (11), 4041–4050. 10.1111/cas.14634 32860283 PMC7648034

[B114] Zhang L. Liu J. Hou Y. (2023). Classification, function, and advances in tsRNA in non-neoplastic diseases. Cell Death Dis. 14 (11), 748. 10.1038/s41419-023-06250-9 37973899 PMC10654580

[B115] Zhang S. Zhao J. Bai X. Handley M. Shan F. (2021). Biological effects of IL-15 on immune cells and its potential for the treatment of cancer. Int. Immunopharmacol. 91, 107318. 10.1016/j.intimp.2020.107318 33383444

[B116] Zhang Y. Guo M. Xin N. Shao Z. Zhang X. Zhang Y. (2016). Decreased microRNA miR-181c expression in peripheral blood mononuclear cells correlates with elevated serum levels of IL-7 and IL-17 in patients with myasthenia gravis. Clin. Exp. Med. 16 (3), 413–421. 10.1007/s10238-015-0358-1 25962782

[B117] Zhao J. L. Rao D. S. Boldin M. P. Taganov K. D. O'Connell R. M. Baltimore D. (2011). NF-kappaB dysregulation in microRNA-146a-deficient mice drives the development of myeloid malignancies. Proc. Natl. Acad. Sci. U. S. A. 108 (22), 9184–9189. 10.1073/pnas.1105398108 21576471 PMC3107319

[B118] Zhong H. Lu J. Jing S. Xi J. Yan C. Song J. (2020). Low-dose rituximab lowers serum Exosomal miR-150-5p in AChR-positive refractory myasthenia gravis patients. J. Neuroimmunol. 348, 577383. 10.1016/j.jneuroim.2020.577383 32961347

[B119] Zhou Y. Yan C. Gu X. Zhou L. Lu J. Zhu W. (2021). Short-term effect of low-dose rituximab on myasthenia gravis with muscle-specific tyrosine kinase antibody. Muscle Nerve 63 (6), 824–830. 10.1002/mus.27233 33745138

[B120] Zhu M. Wang Y. Xu X. Guo X. Mao Y. Gao F. (2024). Small extracellular vesicle microRNAs in pediatric myasthenia gravis plasma and skeletal muscle. Postgrad. Med. J. 100 (1185), 488–495. 10.1093/postmj/qgae015 38449066

[B121] Zhuang J. Guan M. Liu M. Liu Y. Yang S. Hu Z. (2021). Immune-related molecular profiling of thymoma with myasthenia gravis. Front. Genet. 12, 756493. 10.3389/fgene.2021.756493 34777476 PMC8580862

